# Filling Teeth

**Published:** 1878-02

**Authors:** J. B. Tullis


### FILLING TEETH.

BY J. B. TULLIS, D. D. S.

Much has been written and said on the subject of filling teeth. The anxiety of the dentist and desire of the patient, makes this a very important question. The character and skill of the dentist is in jopardy all the time. If he is successful his praise is sounded from the "house top," his failures are borne on every breeze. To make a good filling and preserve the tooth, is the highest ambition of the dentist. Many claim to do this, and many fail to accomplish the task.

To make a good filling, first examine carefully the tooth and the cavity as far as possible at the time. Then decide what is best and proceed to work. Remove all the decay and soft dentine from the cavity, so that it will retain the material. The cavity should be cleaned of all decay, and all chips and bone dust should be removed. Prepare the foil or other material for filling, adjust the rubber dam, or napkin to keep the cavity dry. Be careful not to introduce the foil too rapidly; place it against the wall and press to its place, and condense as you build up, fill out the cavity full, or if you are building out a tooth, let it be free and full, so when finished, it will have the natural form. To do this, there is a variety of modes. Some will say without an engine, rubber dam and mallet you can not make a good filling. That is to say, all fillings put in before these modern improvements, were failures. I have seen some fine work done by hand pressure, using the napkin and no engine, as I have ever seen. In fact, a good many fillings and teeth are hammered to death. The edges of the cavity are frequently fractured by the edge of the plugger and force of the mallet, and in time fail. Amalgam is discarded by many good dentists, and used extensively by others, in fact, it is regarded by some superior to gold in preserving the teeth. I have used gold, amalgam and tin foil for permanent fillings, and have success with all these materials and some failures. I have never reached the point where I never fail, yet our failures should not be too frequent. All cavities to be filled should be prepared with care and the material properly introduced, and condensed and finished, and we would not hear of so many failures.

I find those who confine themselves to a certain line of work, have as many failures as those who are more eclectic in their practice. I do not think one line of practice alone will meet all the cases that occur in daily practice.

